# Supplementary material for: Microfluidic Obstacle Arrays Induce Large Reversible Shape Change in Red Blood Cells
Source: Micromachines (Basel). 2021 Jun 30;12(7):783. doi: 10.3390/mi12070783 (PMC8303182; doi:10.3390/mi12070783)
Supplement: Supplementary file 1 [file micromachines-12-00783-s001.zip › Supplementary Materials.pdf]

Supplementary Materials

MOVIE S1 Movie showing a red blood cell entering the obstacle array. The array has a minimum post separation of 7 μm, 18-μm diameter posts and a row shift fraction of 1/50. The peak cell speed in between the gaps is 18.6 mm/s, corresponding to a peak shear rate of 10,600 s^{-1}. The frame interval is 250 μs. The movie has been slowed 333 times to 12 fps and encoded using H.264 compression.

MOVIE S2 Movie showing a red blood exiting the obstacle array. The array has a minimum post separation of 7 μm, 18-μm diameter posts and a row shift fraction of 1/50. The peak cell speed in between the gaps is 9.6 mm/s, corresponding to a peak shear rate of 5500 s^{-1}. The frame interval is 2.5 ms. The movie has been slowed 33 times to 12 fps and encoded using H.264 compression.

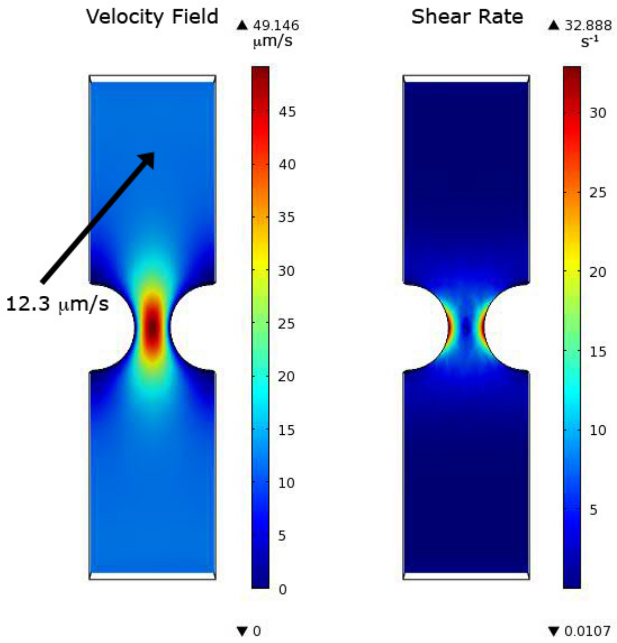

FIGURE S1 Velocity field and shear rate at the middle of the channel in a 3-dimensional steady- state single phase incompressible Navier Stokes model of fluid flow. The section is 100 μm long, 25 μm wide and 32 μm deep. The pressure drop is 0.1 Pa. The gap is 7 μm and the posts have a 9-μm radius. The peak fluid speed in the gap is 49.2 μm/s and the peak fluid speed in the open region is 12.3 μm/s, giving a ratio of 4. The location of peak shear is on the post surface at the gap constriction.
